# Supplementary material for: The RNA-binding protein AKAP8 suppresses tumor metastasis by antagonizing EMT-associated alternative splicing
Source: Nat Commun. 2020 Jan 24;11:486. doi: 10.1038/s41467-020-14304-1 (PMC6981122; doi:10.1038/s41467-020-14304-1)
Supplement: Supplementary file 1 — Supplementary Information [file 41467_2020_14304_MOESM1_ESM.pdf]

SUPPLEMENTARY INFORMATION

The RNA binding protein AKAP8 suppresses tumor metastasis by antagonizing EMT associated alternative splicing

Xiaohui Hu[1,2,4], Samuel E. Harvey[1,2,4], Rong Zheng[1,2], Jingyi Lyu[1,2], Caitlin L. Grzeskowiak[2], Emily Powell[3], Helen Piwnica-Worms[3], Kenneth L. Scott[2], Chonghui Cheng[1,2]*

[1]Lester & Sue Smith Breast Center, Baylor College of Medicine, Houston, TX 77030

[2]Department of Molecular and Human Genetics, Baylor College of Medicine, Houston, TX 77030

[3]Department of Experimental Radiation Oncology, The University of Texas MD Anderson Cancer Center, Houston, TX 77030

[4]These authors contributed equally to this work.

*Corresponding Author. Email: chonghui.cheng@bcm.edu

Supplementary Figure 1

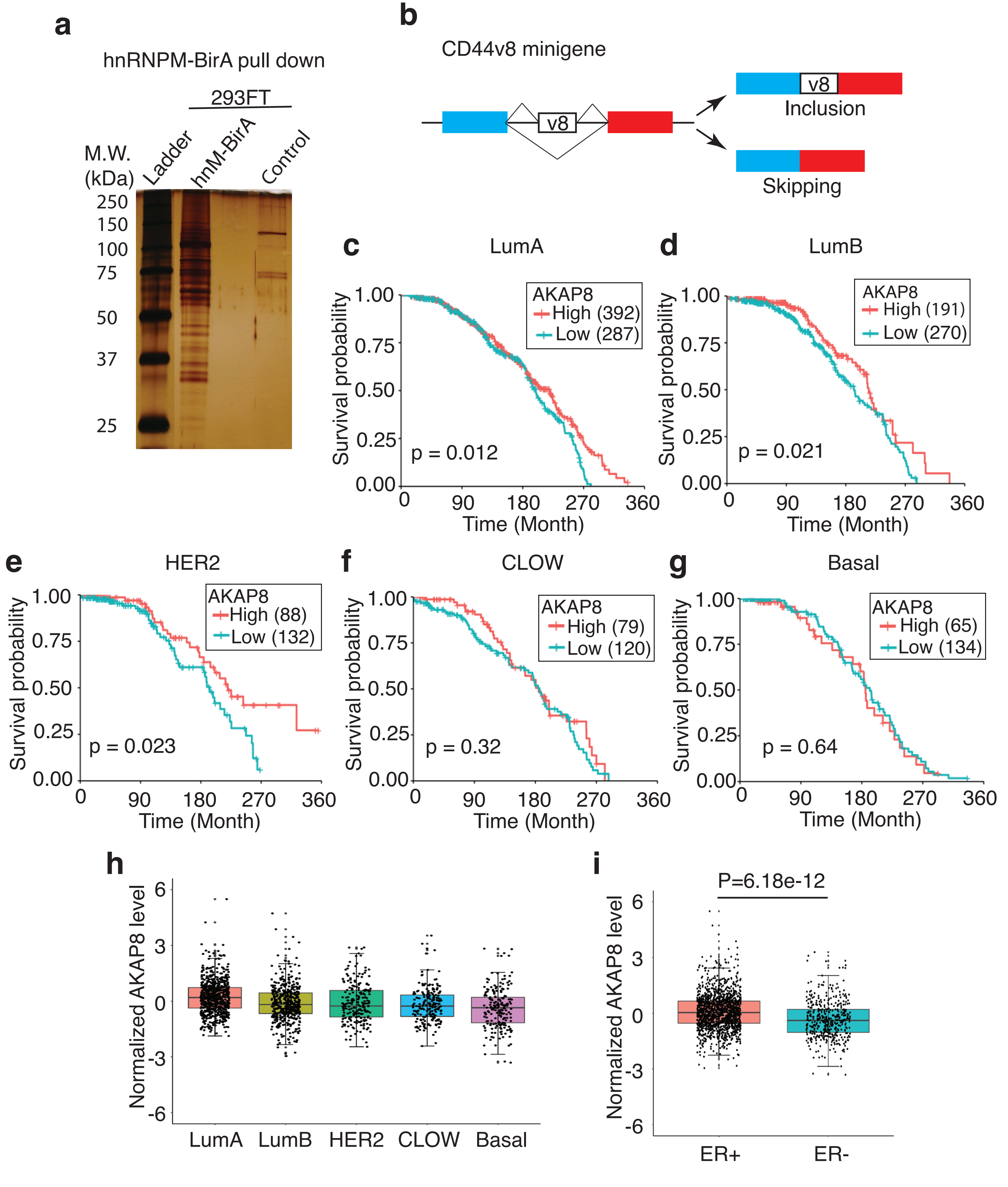

Supplementary Figure 1. Functional screening to identify AKAP8 as an hnRNPM-interacting protein. (**a**) Silver stain image showing BioID pull down band patterns in 293FT cells with or without hnRNPM-BirA expression. (**b**) Schematic of the CD44v8 splicing reporter minigene, with upstream and downstream constitutive exons showing in blue and red rectangles, respectively. (**c-g**) Kaplan Meier plot analysis of AKAP8 expression correlation with patient overall survival in five breast cancer subtypes from the METABRIC breast cancer dataset. (**h, i**) Box and whiskers plots with jitters representing AKAP8 mRNA expression levels across different breast cancer subtype (**h**) and different ER status (**i**) using the breast cancer METABRIC database. The line within each box represents the median. Upper and lower edges of each box represent 75th and 25th percentile, respectively. The whiskers represent the maximum and minimum values within 1.5 x the interquartile range. LumA: Luminal A; LumB: Luminal B; HER2: HER2+; CLOW: Claudin Low. P values in c-i were determined by two sample z-test.

Supplementary Figure 2

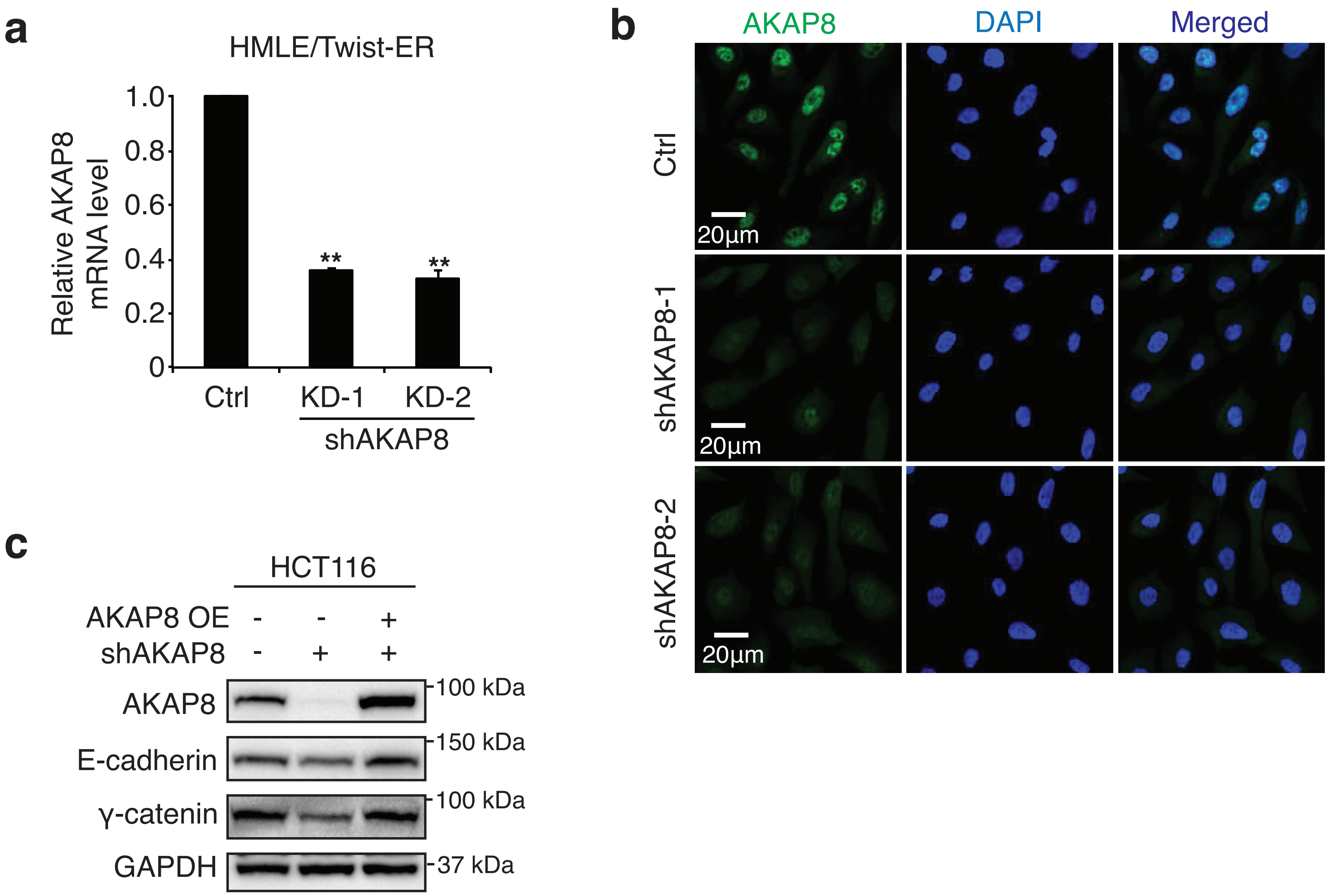

Supplementary Figure 2. Depletion of AKAP8 promotes an EMT phenotype. (a) qRT-PCR analysis of AKAP8 knockdown efficiency in HMLE/Twist-ER cells, relative AKAP8 mRNA levels were normalized to TBP. (**) P < 0.01 between control (Ctrl) and AKAP8 knockdown groups (KD-1, KD-2). Error bars indicate s.d. **(b)** Immunofluorescence analysis for testing AKAP8 expression in control, shAKAP8-1, shAKAP8-2 HMLE/Twist-ER cells. AKAP8 were stained in green, and DAPI were stained in blue. **(c)** Western blot analysis of epithelial markers, E-cadherin and γ-catenin, in HCT116 cells expressing AKAP8 shRNA or AKAP8 shRNA plus AKAP8 cDNA (AKAP8 OE). This AKAP8 cDNA contains three synonymous mutations in the shAKAP8 targeting region. Source data are provided as a Source Data file.

Supplementary Figure 3

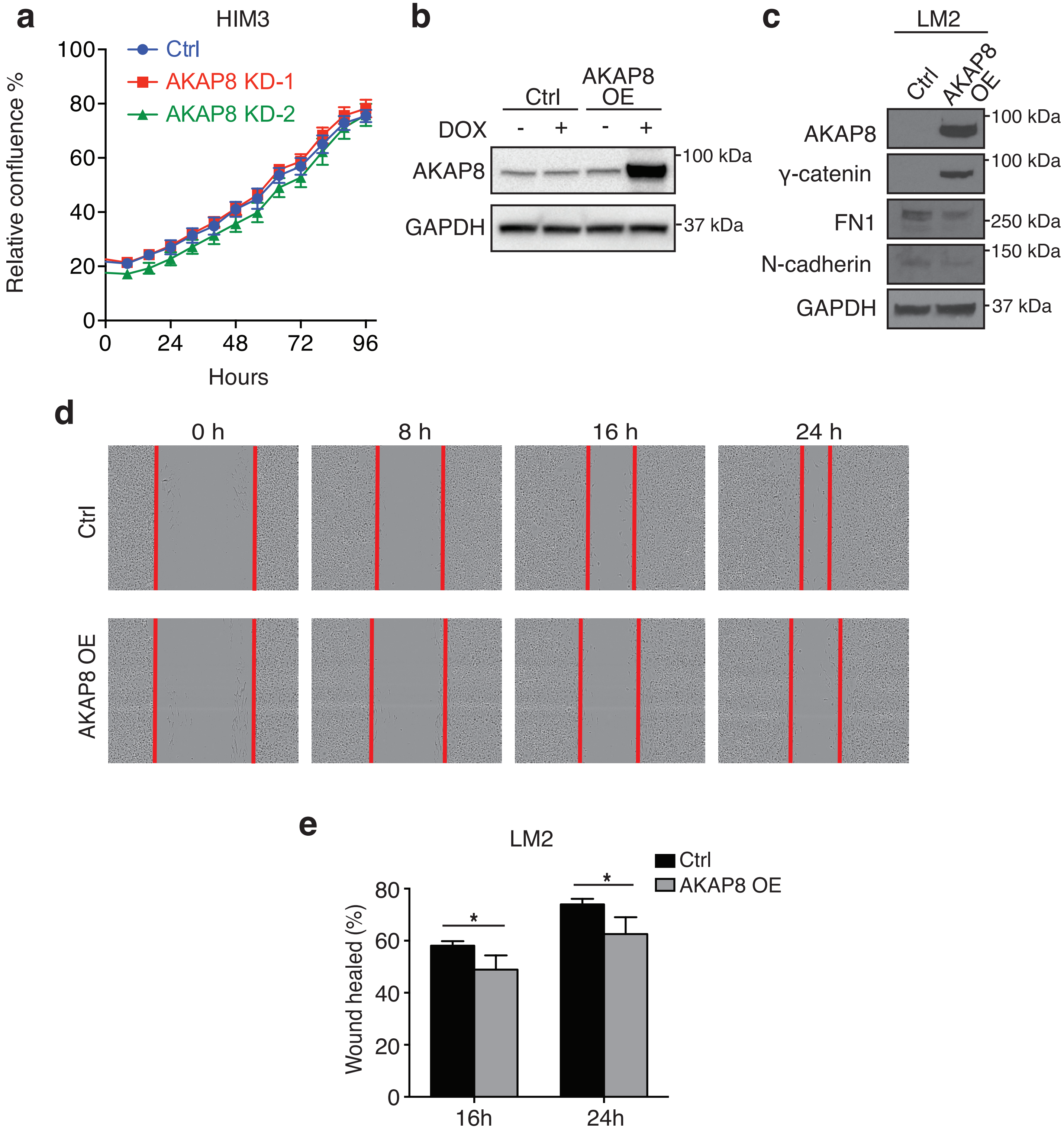

Supplementary Figure 3. AKAP8 suppresses breast cancer metastasis. (**a**) Cell proliferation assay showing that control (Ctrl) and AKAP8 knockdown HIM3 cells proliferate at a similar rate. (**b**) Western blot analysis indicates AKAP8 expression in LM2 cells expressing DOX-inducible control (Ctrl, pCW57) and AKAP8 OE (pCW57-AKAP8) plasmids. (**c**) Western blot analysis showing the increase of epithelial marker, γ-catenin, and the decrease of mesenchymal markers, FN1 and N-cadherin, in LM2 cells overexpressing AKAP8. (**d**) Representative phase-contrast images showing the wound healing images of control and AKAP8 OE LM2 cells at indicated time points. (**e**) Bar graphs showing the quantification of wound healing efficiency in panel d. (*) $P<0.05$. P value was tested by Student's T-test, two-tailed. n = 3. All error bars indicate s.d. Source data are provided as a Source Data file.

Supplementary Figure 4

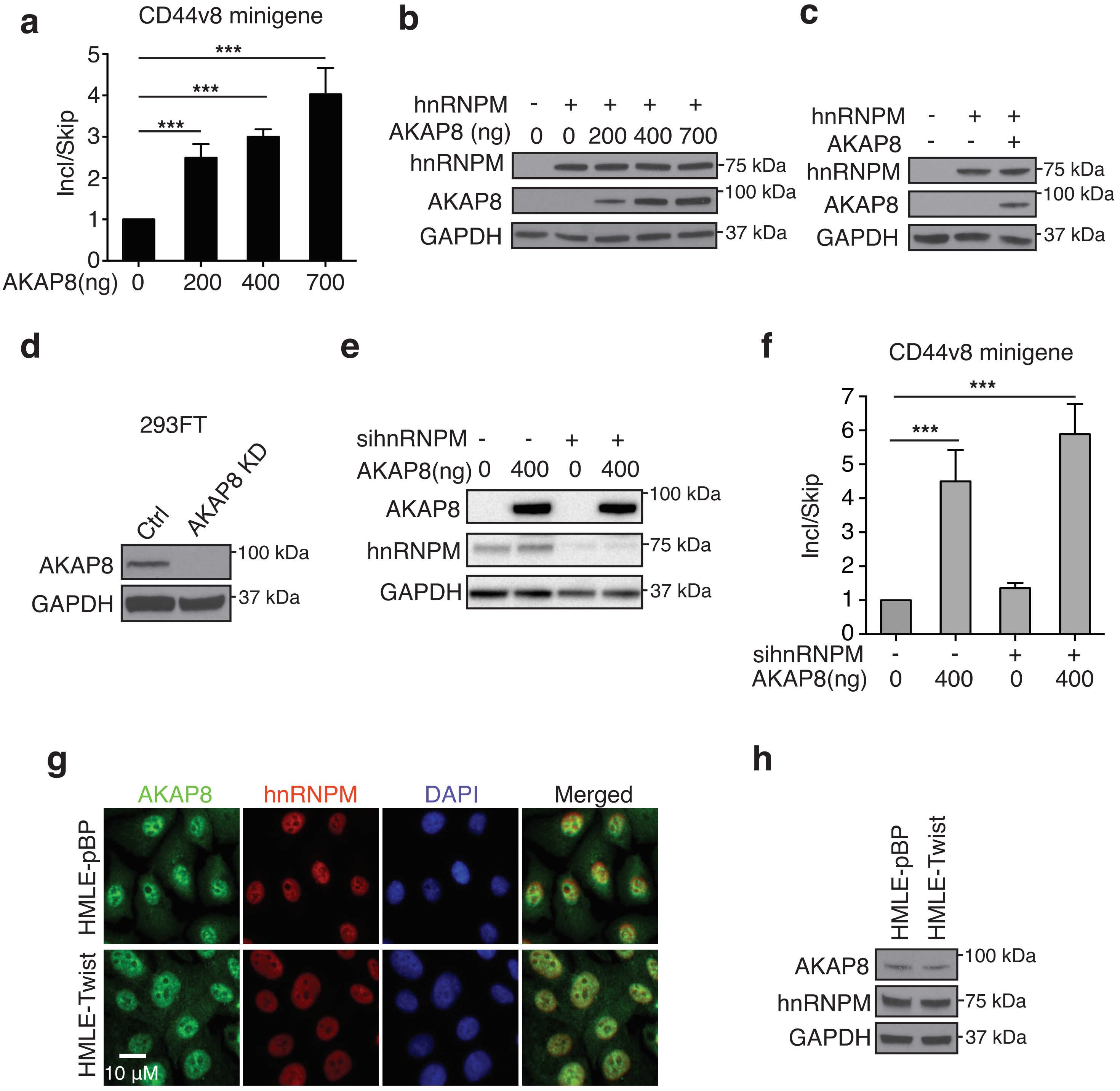

Supplementary Figure 4. AKAP8 antagonizes hnRNPM's splicing activity. (**a**) qRT-PCR analysis of CD44v8 splicing minigene assay indicating that AKAP8 promotes v8 inclusion in a dose-dependent manner. (***) P<0.001. (**b, c**) Western blot analysis of hnRNPM and AKAP8 in experiments described in Fig. 4a and Fig. 4c. (**d**) Western blot analysis of AKAP8 knockdown efficiency in 293FT cells. (**e, f**) CD44v8 minigene assay showing AKAP8 splicing activity in control and sihnRNPM 293FT cells. Western blot analysis of AKAP8 and hnRNPM expression (**e**) and qRT-PCR analysis of CD44v8 minigene splicing (**f**) are shown. (***) P<0.001. (**g**) Immunofluorescence staining showing no observed differences on the colocalization of AKAP8 and hnRNPM in epithelial HMLE-pBP and mesenchymal HMLE-Twist cells. (**h**) Western blot analysis showing AKAP8 and hnRNPM expression levels in epithelial HMLE-pBP and mesenchymal HMLE-Twist cells. P value was tested by Student's T-test, two-tailed. All error bars indicate s.d., n = 3. Source data are provided as a Source Data file.

Supplementary Figure 5

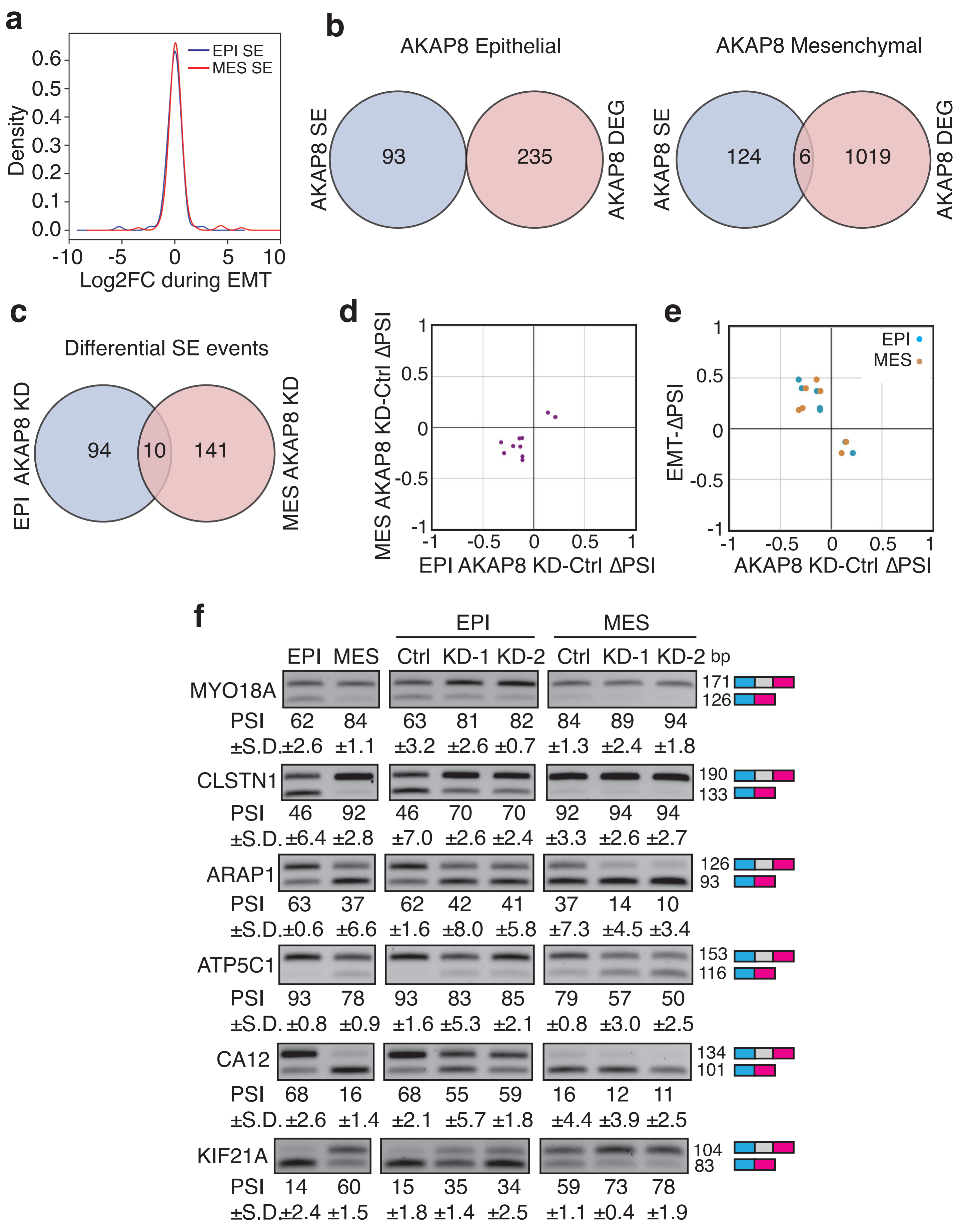

Supplementary Figure 5. AKAP8 regulates EMT in a cell-state specific manner through alternative splicing. (**a**) Kernel Density Estimate gene expression plot of genes that undergo significant skipped exon (SE) in epithelial (EPI, blue line) and mesenchymal (MES, red line) cells, showing that no significant gene expression changes were observed. (**b**) Venn diagram indicating the overlapping of AKAP8 regulated SE and DEG (differentially expressed genes) in both epithelial (Left) and mesenchymal (Right) cells. (**c**) Venn diagram showing overlapping SEs that are regulated by AKAP8 in epithelial (EPI) and mesenchymal (MES) cells. (**d**) Scatter plot showing the ∆PSI values of the 10 shared splicing events regulated by AKAP8 in epithelial and mesenchymal states. PSI: Percent Spliced In. (**e**) Scatter plot showing the ∆PSI values of the AKAP8 regulated seven shared splicing events during EMT. (**f**) Semi-qPCR analysis of splicing events regulated during EMT that was also regulated by AKAP8. Source data are provided as a Source Data file.

Supplementary Figure 6

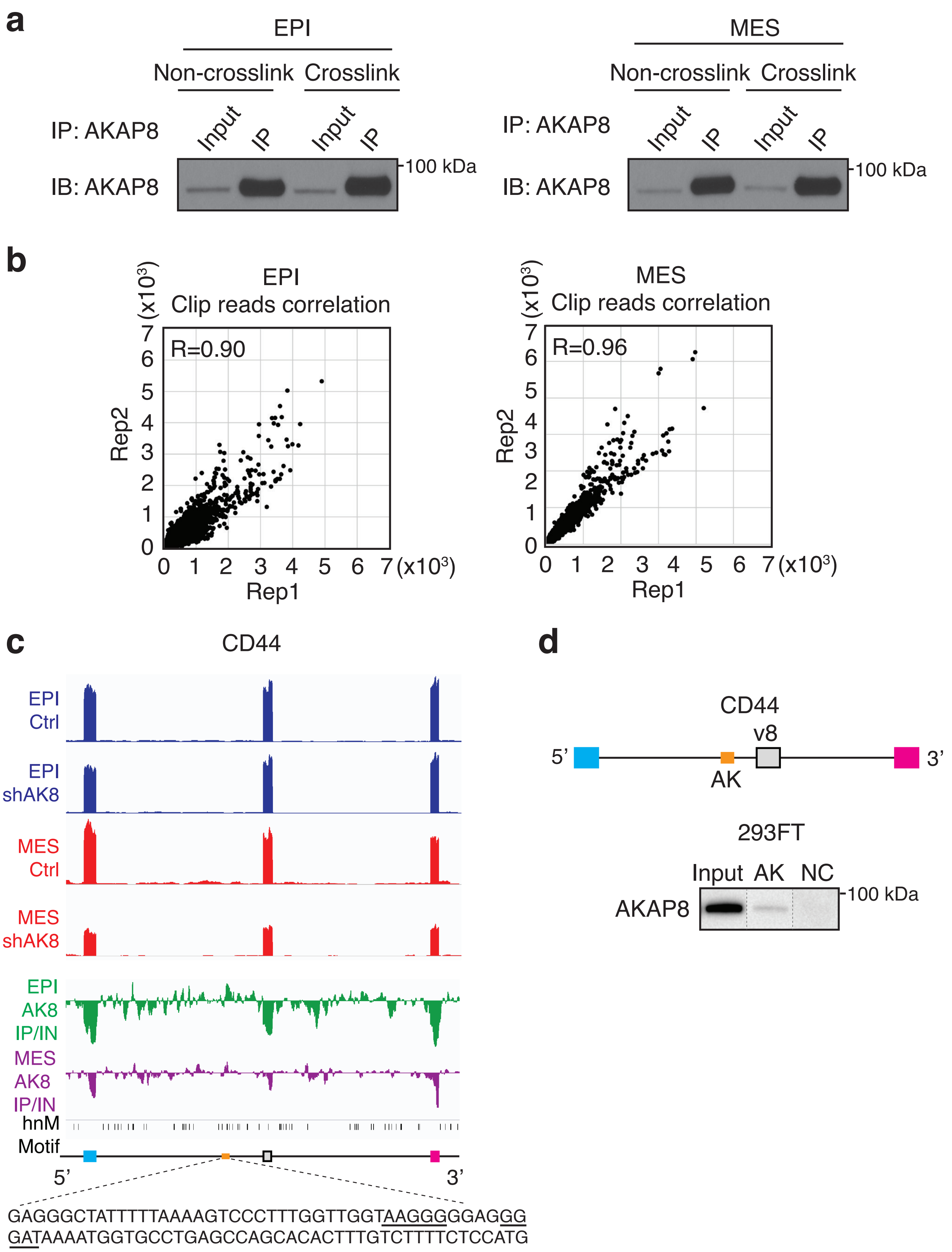

Supplementary Figure 6. AKAP8 eCLIP identifies its RNA binding targets and cis-element motifs. (**a**) Western blot analysis for IP-ed AKAP8 in both crosslinked and non-crosslinked HMLE/Twist-ER cells in the epithelial cell state (EPI, left panel) and the mesenchymal cell state (MES, right panel). (**b**) Scatterplots showing eCLIP reads correlation between replicates (Rep1 and Rep2) in the epithelial cell state (EPI, left panel) and the mesenchymal cell state (MES, right panel). (**c**) Integrated genome viewer tracks indicated CD44 v8 exon and flanking introns. Top four tracks represent autoscaled RPM-normalized RNA seq reads. Bottom two tracks represent IP/Input normalized AKAP8 eCLIP signal. Bottom cartoon shows CD44 v8 exon in light grey flanked by upstream (blue) and downstream (pink) exons. Black bricks indicate hnRNPM (hnM) binding locations. Yellow bar indicates AKAP8 eCLIP binding site. Zoomed out sequence indicates nucleotide sequence covered by binding site with G stretches > 3 nucleotides underlined. (**d**) Top panel, a schematic of the CD44 v8 pre-mRNA and the cis-element sequences are shown. The AKAP8 eCLIP binding peak in (**c**) is denoted as AK and is shown in orange. Bottom panel, western blot analysis of AKAP8 binding signal to the AK oligos by RNA pulldown assay. NC: negative control. Source data are provided as a Source Data file.

Supplementary Figure 7

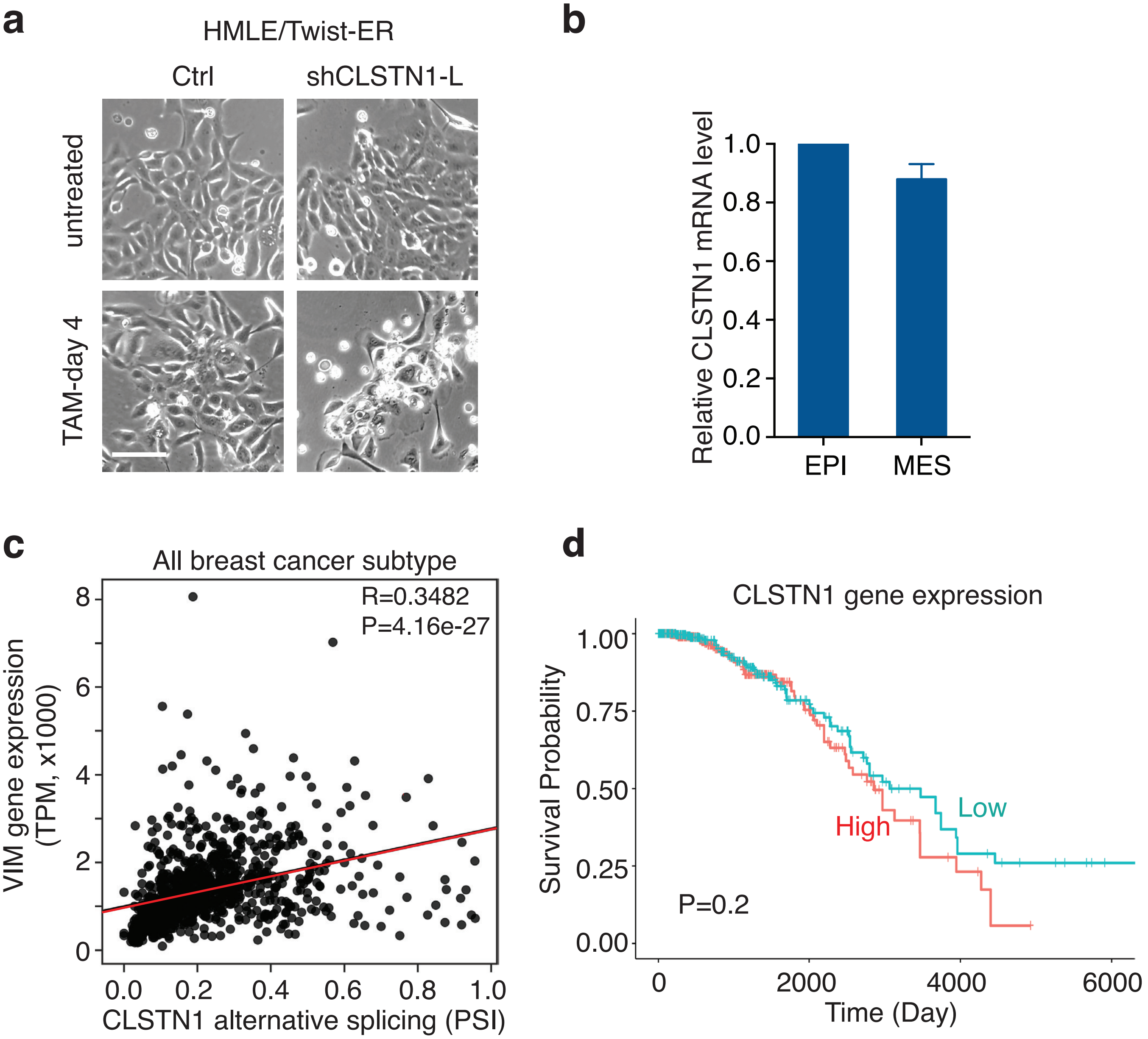

Supplementary Figure 7. The CSLTN1 isoform with exon 11 inclusion promotes EMT. (a) Phase-contrast images (10X) showing a cell death phenotype in control (Ctrl) and CLSTN1-L knockdown cells after 4 days of tamoxifen (TAM) induction. White line represents scale bar at 100 μm. **(b)** qRT-PCR analysis of CLSTN1 relative mRNA level showing that CLSTN1 expression levels were not significantly altered in epithelial (EPI) and mesenchymal (MES) cell states. Error bars represent s.d., n = 3. **(c)** Plot of the mesenchymal marker vimentin (VIM) expression and CLSTN1 alternative splicing, represented by PSI (Percent Spliced In) values, in the breast cancer TCGA dataset showing a significant positive correlation in all breast cancer subtypes. P value was determined by person correlation test. **(d)** Kaplan Meier plot showing that no significant predictions between CLSTN1 gene expression and breast cancer patient survival in TCGA dataset. P value was determined by log rank test. Source data are provided as a Source Data file.

Supplementary Table 1

**Primer Sequences**	Forward	Reverse
**Cloning**		
Cloning hnRNPM to pQCXIP-BirA	TGCAGGAATTGATCCGCGGCCGCATGGC GGCAGGGGTC	CACGGTGTTGTCCTTGGATCCAGC GTTTCTATCAATTCGAACGTC
Cloning AKAP8 to pCDH or pCW57	ATGGACCAGGGCTACGGAG	TTCTGTGGGAACAGCGTCTTTAG
pcw57_AKAP8_mut	ATCAACGTTTGGACATGATGTCCAAG	TAATCTTGGCAATGAGGGAGTC
**qPCR**		
AKAP8	GAAGCAGTTCCAACTTTACGAGG	CAGAGTTCATCCTCACCCTTG
hnRNPM	GAGCCATATGCCAATCCAAC	AGCGTCCATTAAGAGCTCCA
ESRP1	CAGAGGCACAAACATCACAT	AGAAACTGGGCTACCTCATTGG
RBFOX2	TTTAATGAACGTGGCTCTAAGGG	CGGACCATATACAGCTCCAAC
QKI	CTGATGCTGTGGGACCTATTG	GTTGTTTGGCTGTAAGTCCTCT
CD44v8 minigene inclusion	CAATGACAACGCTGGCACAA	CCAGCGGATAGAATGGCGCCG
CD44v8 minigene skipping	GAGGGATCCGGTTCCTGCCCC	CAGTTGTGCCACTTGTGGGT
CD44v5 minigene inclusion	GAGGGATCCGGTTCCTGCCCC	TGCTTGTAGAATGTGGGGTCT
CD44v5 minigene skipping	GAGGGATCCGGTTCCTGCCCC	CAGTTGTGCCACTTGTGGGT
CLSTN1 total	AACAGATATGAATCGGCACCAC	CCATTCCTCATCACAGACCTG
**semi-PCR**		
CD44v8/v5 minigene	GAGGGATCCGGTTCCTGCCCC	CCAGCGGATAGAATGGCGCCG
CLSTN1	GTGACTGAGGATTACCCGCTC	CGGAACGGAGAGTTAAGCCAG
ESYT2	TGCTAACAGACATCAAAGCTGAC	TTGCTCTCCTGGGCCTTGTG
MYO18A	GCTGCCATTGAGGATGAGATG	GTTTTTTGACAACCAGGACTTGAC
ARAP1	GCTACTTCATCCTCAACAGCAG	CTTTGAGACTCTTAATAGGCCACTC
ATP5C1	CGCCAAGCTGTCATCACAAAAG	TCGGACAAAGGCAGCAGTAAG
CA12	GCAGGTCCAGAAGTTCGATGAG	CCACAATACAGATGCCAAGAATG
KIF21A	GAAGGCAGAATTAAATCCTGAGCTAG	CATCCTCATCAGTACTATCCTCTAC
**shRNA Seqeunces**		
shRNA	sense	antisense
shAKAP8-1	GCCAAGATCAACCAGCGTTTG	CAAACGCTGGTTGATCTTGGC
shAKAP8-2	GCTGAAGTACATTGTCCTTAG	CTAAGGACAATGTACTTCAGC
shCLSTN1 long	CAGGAGTTGAAAATGACAATG	CATTGTCATTTTCAACTCCTG
shCLSTN1 short	GCTTGCTGGCAAGGTGGCGAC	GTCGCCACCTTGCCAGCAAGC
hnRNPM siRNA	ordered from ThermoFisher, ID: s9261	

Supplementary Table 2

Alternative Splicing Cutoffs

cstat	0.01
FDR	0.1
dPSI	0.05
Reads	10
FPKM	5

eCLIP Cutoffs

log2FC IP/Input	1
adj p-value	0.05
region	+/- 1000 bp from variable exons

AKAP8 Epithelial SE

Gene Name	Splice ID	Splicing Direction	Upstream Binding Site	Downstream Binding Site
FBXO18	SE:chr10:5932981-5933079:5932242-5932309:5944982-5945138:+:FBXO18	Inclusion	Yes	No
CCDC85C	SE:chr14:99983424-99983520:99982524-99982623:99988469-99988577:-:CCDC85C	Inclusion	Yes	No
MZF1	SE:chr19:59083916-59084086:59082566-59082796:59084421-59084678:-:MZF1	Inclusion	Yes	No
TSPO	SE:chr22:43555215-43555426:43547519-43547610:43557057-43557196:+:TSPO	Inclusion	Yes	No
GAK	SE:chr4:898424-898567:891820-891946:907394-907456:-:GAK	Inclusion	Yes	No
CARS	SE:chr11:3068982-3069231:3063394-3063486:3078572-3078667:-:CARS	Inclusion	No	Yes
TPD52L2	SE:chr20:62507168-62507228:62505020-62505169:62514071-62514173:+:TPD52L2	Inclusion	No	Yes
APEH	SE:chr3:49711921-49712054:49711434-49711846:49713126-49713220:+:APEH	Inclusion	No	Yes
CLSTN1	SE:chr1:9797555-9797612:9795942-9796100:9801151-9801314:-:CLSTN1	Skipping	Yes	No
CALML4	SE:chr15:68491878-68492019:68489777-68489966:68497458-68497489:-:CALML4	Skipping	Yes	No
SH2B1	SE:chr16:28877332-28878354:28874988-28875075:28878694-28878753:+:SH2B1	Skipping	Yes	No
BCL2L11	SE:chr2:111881626-111881716:111881309-111881446:111907620-111907724:+:BCL2L11	Skipping	Yes	No
SEPT2	SE:chr2:242256913-242257014:242255296-242255397:242263823-242264659:+:SEPT2	Skipping	Yes	No
MICALL2	SE:chr7:1473845-1474783:1468254-1468427:1476377-1476492:-:MICALL2	Skipping	Yes	Yes
ENTPD6	SE:chr20:25201867-25201969:25199185-25199250:25203473-25203606:+:ENTPD6	Skipping	No	Yes
PUF60	SE:chr8:144902835-144902886:144900542-144900704:144903766-144903856:-:PUF60	Skipping	No	Yes

AKAP8 Mesenchymal SE

Gene Name	Splice ID	Splicing Direction	Upstream Binding Site	Downstream Binding Site
TSPAN4	SE:chr11:847200-847300:842807-842915:850287-850367:+:TSPAN4	Inclusion	Yes	Yes
CARS	SE:chr11:3068982-3069231:3063394-3063486:3078572-3078667:-:CARS	Inclusion	Yes	No
RBM14-RBM4	SE:chr11:66407170-66407594:66384101-66384528:66413497-66413940:+:RBM14-RBM4	Inclusion	Yes	No
PKD1	SE:chr16:2166833-2167054:2166529-2166645:2167489-2167673:-:PKD1	Inclusion	Yes	No
BOK	SE:chr2:242501762-242501891:242498135-242498408:242509539-242509703:+:BOK	Inclusion	Yes	Yes
RRBP1	SE:chr20:17660643-17660720:17639240-17641173:17662672-17662705:-:RRBP1	Inclusion	Yes	No
PISD	SE:chr22:32019669-32019835:32017634-32017871:32044086-32044262:-:PISD	Inclusion	Yes	No
EIF4A2	SE:chr3:186502750-186502890:186502352-186502485:186503671-186503702:+:EIF4A2	Inclusion	Yes	No
CTBP1	SE:chr4:1235112-1235307:1221986-1222131:1242703-1242764:-:CTBP1	Inclusion	Yes	No
CCND3	SE:chr6:41908107-41908323:41904592-41905132:42016238-42016414:-:CCND3	Inclusion	Yes	No
NEK6	SE:chr9:127055127-127055292:127020241-127020364:127064214-127064333:+:NEK6	Inclusion	Yes	Yes
INF2	SE:chr14:105181620-105181677:105180539-105181193:105185131-105185942:+:INF2	Inclusion	No	Yes
SUGP2	SE:chr19:19104456-19104549:19101698-19101958:19105174-19106089:-:SUGP2	Inclusion	No	Yes
STK25	SE:chr2:242447420-242447550:242440154-242440211:242447857-242447971:-:STK25	Inclusion	No	Yes
TPD52L2	SE:chr20:62507168-62507228:62505020-62505169:62514071-62514173:+:TPD52L2	Inclusion	No	Yes
PLXNB2	SE:chr22:50733147-50733207:50728528-50729026:50745981-50746032:-:PLXNB2	Inclusion	No	Yes
SLC16A3	SE:chr17:80194604-80194748:80191562-80194107:80218774-80219005:+:SLC16A3	Skipping	Yes	Yes
GSN	SE:chr9:124062333-124062404:124044716-124044828:124064240-124064445:+:GSN	Skipping	Yes	No
BOK	SE:chr2:242509539-242509703:242498135-242498408:242511711-242513553:+:BOK	Skipping	No	No
ARHGEF10	SE:chr8:1828213-1828330:1824736-1824900:1830800-1830915:+:ARHGEF10	Skipping	No	No
