# Supplementary material for: The RNA-binding protein AKAP8 suppresses tumor metastasis by antagonizing EMT-associated alternative splicing
Source: Nat Commun. 2020 Jan 24;11:486. doi: 10.1038/s41467-020-14304-1 (PMC6981122; doi:10.1038/s41467-020-14304-1)
Supplement: Supplementary file 3 — Description of Additional Supplementary Files [file 41467_2020_14304_MOESM3_ESM.pdf]

Description of Additional Supplementary Files

File Name: Supplementary Data 1

Description: Mass Spec results of hnRNPM Bio-ID showing the top 50 hnRNPM-interacting proteins based on their unique peptide counts.
