# Supplementary material for: The RNA-binding protein AKAP8 suppresses tumor metastasis by antagonizing EMT-associated alternative splicing
Source: Nat Commun. 2020 Jan 24;11:486. doi: 10.1038/s41467-020-14304-1 (PMC6981122; doi:10.1038/s41467-020-14304-1)
Supplement: Supplementary file 5 — Reporting Summary [file 41467_2020_14304_MOESM5_ESM.pdf]

nature research

Corresponding author(s): Chonghui Cheng

Last updated by author(s): Dec 14, 2019

Reporting Summary

Nature Research wishes to improve the reproducibility of the work that we publish. This form provides structure for consistency and transparency in reporting. For further information on Nature Research policies, see Authors & Referees and the Editorial Policy Checklist .

Statistics

For all statistical analyses, confirm that the following items are present in the figure legend, table legend, main text, or Methods section.

n/a	Confirmed	
☐	☒	The exact sample size (*n*) for each experimental group/condition, given as a discrete number and unit of measurement
☐	☒	A statement on whether measurements were taken from distinct samples or whether the same sample was measured repeatedly
☐	☒	The statistical test(s) used AND whether they are one- or two-sided<br>*Only common tests should be described solely by name; describe more complex techniques in the Methods section.*
☒	☐	A description of all covariates tested
☐	☒	A description of any assumptions or corrections, such as tests of normality and adjustment for multiple comparisons
☐	☒	A full description of the statistical parameters including central tendency (e.g. means) or other basic estimates (e.g. regression coefficient) AND variation (e.g. standard deviation) or associated estimates of uncertainty (e.g. confidence intervals)
☐	☒	For null hypothesis testing, the test statistic (e.g. *F*, *t*, *r*) with confidence intervals, effect sizes, degrees of freedom and *P* value noted<br>*Give P values as exact values whenever suitable.*
☒	☐	For Bayesian analysis, information on the choice of priors and Markov chain Monte Carlo settings
☒	☐	For hierarchical and complex designs, identification of the appropriate level for tests and full reporting of outcomes
☐	☒	Estimates of effect sizes (e.g. Cohen's *d*, Pearson's *r*), indicating how they were calculated

Our web collection on statistics for biologists contains articles on many of the points above.

Software and code

Policy information about availability of computer code

Data collection	Data were collected on Illumina HiSeq 4000 at the University of Chicago Genomics Facility
Data analysis	RNA-seq reads were aligned to the human genome (GRCh37, primary assembly) and transcriptome (Gencode version 24 backmap 37 comprehensive gene annotation) using STAR version 2.6.1a. Differential alternative splicing was quantified using rMATS version 4.0.2. Differential gene expression analysis was performed by counting reads over genes from the same annotation as alignment using featureCounts version 1.5.0. Differential gene expression analysis was conducted using DESeq2 performed on genes with read abundance larger than 10 counts over the smallest library size of all samples analyzed. eCLIP data processing was conducted using the public eCLIP pipeline version 0.2.1a (https://github.com/YeoLab/eclip/releases/tag/0.2.1a) and public merge-peaks pipeline version 0.0.6 (https://github.com/YeoLab/merge_peaks/ releases/tag/0.0.6), derived from a previously published eCLIP pipeline. Data visualization and statistical analyses were conducted in Python and R.

For manuscripts utilizing custom algorithms or software that are central to the research but not yet described in published literature, software must be made available to editors/reviewers. We strongly encourage code deposition in a community repository (e.g. GitHub). See the Nature Research guidelines for submitting code & software for further information.

Data

Policy information about availability of data

All manuscripts must include a data availability statement. This statement should provide the following information, where applicable:

- Accession codes, unique identifiers, or web links for publicly available datasets
- A list of figures that have associated raw data
- A description of any restrictions on data availability

High throughput sequencing data and differential analysis tables have been uploaded to the GEO database under accession number GSE139074

Field-specific reporting

Please select the one below that is the best fit for your research. If you are not sure, read the appropriate sections before making your selection.

☒ Life sciences ☐ Behavioural & social sciences ☐ Ecological, evolutionary & environmental sciences

For a reference copy of the document with all sections, see nature.com/documents/nr-reporting-summary-flat.pdf

Life sciences study design

All studies must disclose on these points even when the disclosure is negative.

Sample size	No statistical methods were used to pre-determine sample size. The sample size of each experiment is provided in the figure/table legends in the main manuscript and supplementary file. For RNA-seq or eCLIP-seq, samples were prepared at least in two biological replicates. For in vivo mouse model, each group has at least 5 mice. For experiments with live cells, each plots were repeated at least twice with similar results. These sizes have previously been shown as sufficiently powered to determine statistical differences in mean values of our investigated parameters.
Data exclusions	No data was excluded from this study.
Replication	The replication number is indicated in the legend of corresponding figures where applicable. All attempts at replication were successful.
Randomization	All the control and experimental group of mice/cells were grown under identical conditions. No randomization were used.
Blinding	All the control and experimental group of mice/cells were grown under identical conditions. No blinding were used.

Reporting for specific materials, systems and methods

We require information from authors about some types of materials, experimental systems and methods used in many studies. Here, indicate whether each material, system or method listed is relevant to your study. If you are not sure if a list item applies to your research, read the appropriate section before selecting a response.

Materials & experimental systems

n/a	Involved in the study	
☐	☒	Antibodies
☐	☒	Eukaryotic cell lines
☒	☐	Palaeontology
☐	☒	Animals and other organisms
☒	☐	Human research participants
☒	☐	Clinical data

Methods

n/a	Involved in the study	
☒	☐	ChIP-seq
☒	☐	Flow cytometry
☒	☐	MRI-based neuroimaging

Antibodies

Antibodies used	Anti-AKAP8 (Abcam, ab72196); Anti-hnRNPM (Origene technologies, TA301557); Anti-Flag (Sigma, F1804); Anti-GAPDH (EMD Millipore, MAB374); Anti-β-actin (Sigma, A5441); Anti-E-cadherin (Cell Signaling, 3195); Anti-γ-catenin (Cell Signaling, 2309); Anti-Occludin(Abcam, ab168986); Anti-FN1 (BD, 610077); Anti-N-cadherin (BD, 610920); Anti-hnRNPF (Santa Cruz, sc-390048); Anti-RBMX (Cell signaling, 14794); Anti-RBM10 (One World Lab); Anti-hnRNPR (One World Lab); Anti-PTBP1 (One World Lab).
Validation	All the antibodies tested in the manuscript has been published or described previously. Their original links are listed in the below:<br>Anti-AKAP8: https://www.abcam.com/akap-95-antibody-ab72196.html;<br>Anti-hnRNPM: https://www.origene.com/catalog/antibodies/primary-antibodies/ta301557/hnrpm-hnrnpm-mouse-monoclonal-antibody-clone-id-2a6;<br>Anti-Flag: https://www.sigmaaldrich.com/catalog/product/sigma/f1804?lang=en&region=US;<br>Anti-GAPDH: https://www.antibodypedia.com/gene/3923/GAPDH/antibody/554563/MAB374;<br>Anti-β-actin: https://www.sigmaaldrich.com/catalog/product/sigma/a5441?lang=en&region=US&gclid=CjwKCAiA5JnuBRA-EiwA-0ggPcybQ4yJbITj1buMC6PsFVtbw_1YT7gHotVT4xHw0JR9l2mTy4eCUxoCu5gQAvD_BwE;<br>Anti-E-cadherin (Cell Signaling, 3195): https://www.cellsignal.com/products/primary-antibodies/e-cadherin-24e10-rabbit-mab/3195;<br>Anti-γ-catenin: https://www.cellsignal.com/products/primary-antibodies/g-catenin-antibody/2309;<br>Anti-Occludin: https://www.abcam.com/occludin-antibody-ab168986.html;<br>Anti-FN1: https://www.bdbiosciences.com/eu/applications/research/stem-cell-research/cancer-research/human/purified-mouse-anti-fibronectin-10fibronectin/p/610077;<br>Anti-N-cadherin: https://www.bdbiosciences.com/us/applications/research/stem-cell-research/cancer-research/human/purified-

mouse-anti-n-cadherin-32n-cadherin/p/610920;
Anti-hnRNPF: https://www.scbt.com/p/hnrnp-f-h-antibody-b-10
Anti-RBMX: https://www.cellsignal.com/products/primary-antibodies/rbmx-hnrnp-g-d7c2v-rabbit-mab/14794?Ns=product.sortId%7C0&N=4294956287&Ntt=motif&Nrpp=30&No=120&fromPage=plp;

Eukaryotic cell lines

Policy information about cell lines

Cell line source(s)	293FT(ATCC); HMLE(From Dr. Robert Weinberg at the Whitehead Institute, MIT); HMLE/Twist-ER(from Dr. Jing Yang at UCSD); HCT116(ATCC); HCT116 (ATCC); HIM3(provided by Dr. Helen Piwnica-Worms at MD Anderson); LM2 (from Dr. Yibin Kang at Princeton University)
Authentication	Cell lines were not authenticated
Mycoplasma contamination	All cell lines tested are negative for mycoplasma.
Commonly misidentified lines (See ICLAC register)	None used.

Animals and other organisms

Policy information about studies involving animals; ARRIVE guidelines recommended for reporting animal research

Laboratory animals	We used 6 to 8 weeks old female NSG nude mice for tumor lung metastasis model by tail vein injection in this study.
Wild animals	This study didn't involve wild animals.
Field-collected samples	This study didn't involve field-collected samples.
Ethics oversight	All animal procedures were performed in accordance with IACUC-approved protocols at Baylor College of Medicine.

Note that full information on the approval of the study protocol must also be provided in the manuscript.
